# The Effect of Dexmedetomidine Sedation on Lower Gastrointestinal Motility in Children—Is It Suitable for Anorectal Manometry?

**DOI:** 10.3390/jcm12237494

**Published:** 2023-12-04

**Authors:** Tal David Berger, Karina Lukovits, David Cavanaugh, Samuel Nurko, Keira Mason

**Affiliations:** 1 Department of Gastroenterology, Boston Children’s Hospital, Boston, MA 02115, USA; taldavid.berger@childrens.harvard.edu (T.D.B.); samuel.nurko@childrens.harvard.edu (S.N.); 2 Geisel School of Medicine, Dartmouth College, Hanover, NH 03755, USA; karina.lukovits@gmail.com; 3 Boston Biostatistical Consulting, North Reading, Haverhill, MA 01832, USA; dmcav6@gmail.com; 4 Department of Anaesthesia, Critical Care and Pain Medicine, Boston Children’s Hospital, Boston, MA 02115, USA

**Keywords:** children, constipation, dexmedetomidine, manometry, outcomes, sedation

## Abstract

Anorectal manometry is one of the most frequently performed gastrointestinal motility studies in children. It is an important study in diagnosing Hirschsprung disease (HD). These procedures can be uncomfortable, painful and emotionally distressing. Nitrous oxide or midazolam are the only pharmacologic options available, as clinical experience suggests that they do not alter manometry readings. Our study was designed to determine whether Dexmedetomidine (DEX) could provide adequate sedation without disrupting anal and rectal pressure. The effect of DEX on anorectal function has never been studied in children. This prospective study recorded anorectal manometry (ARM) measurements prior to the administration of DEX and then repeated the measurements at 1 and 5 min after DEX. The main ARM measurements included resting intra-anal sphincter pressure (IASP) and the presence and characteristics of the recto-anal inhibitory reflex (RAIR). DEX was administered as a bolus followed by a continuous infusion. Twenty patients were included (60% female; mean age 10.8 ± 4.6 years). The RAIR became absent in 2/16 (12.5%) patients after DEX administration. DEX may alter physiologic ARM and IASP recordings necessary to diagnose gastrointestinal medical conditions.

## 1. Introduction

Anorectal manometry (ARM) is the most common motility study performed in children, and is frequently used as part of the diagnostic workup for children with chronic severe constipation in order to evaluate the neuromuscular function of the rectum and anus [1,2]. There are many indications for anorectal manometry. These include anorectal symptoms (chronic constipation, fecal incontinence, and fecal urgency), spinal cord malformation, anorectal malformations, neuromuscular disorders, rectal trauma, suspected Hirschsprung disease cases, and evaluation of constipation in post-surgical Hirschsprung disease patients. Anorectal manometry can also assist with the selection of a specific and appropriate treatment, such as an injection of botulinum toxin to the anal sphincter or use of biofeedback therapy [2]. ARM is performed by inserting a catheter into the anal canal, which is capable of measuring pressure in the anal canal. There are two types of anorectal manometry equipment: A conventional manometry that uses only a few sensors to measure the pressure in the anal canal and presents these measurements as line traces, and a high-resolution manometry, that uses many closely spaced sensors to perform the measurements, and then present the results in a more detailed manner, as a pressure map of the anorectum [2].

The anorectal manometry can provide information on anal sphincter function, defecation dynamics, rectal sensation, and the presence of recto-anal inhibitory reflex (RAIR). The RAIR is a physiological involuntary reflex elicited by the distension of the rectum wall, which is then followed by a decrease in internal anal sphincter pressure (IASP). This relaxation occurs when the myenteric postganglionic parasympathetic neurons release nitric oxide and VIP [2], and this release can be elicited with the distention of a balloon in the rectum, such as during ARM. When there is an absence of ganglion cells in the intestine, such as in patients with Hirschsprung’s disease, there is a lack of relaxation of the sphincter which results in obstruction in the distal colonic. A lack of relaxation can be exhibited during ARM by performing balloon distention. Therefore, the presence or absence of RAIR is a key element in the diagnosis of abnormal non-relaxing internal anal sphincter disorders, such as Hirschsprung disease (HD) and anal sphincter achalasia [1,2,3,4,5,6,7]. ARM allows one to assess other characteristics of anorectal function in addition to RAIR. It is most useful clinically if the patients are awake, able to follow commands, and without any sedatives that may abnormally alter ARM recordings, in order to best simulate defecation. In clinical practice, the main indication for ARM in pediatric patients with constipation is to establish the presence of absence of the RAIR, and therefore exclude Hirschsprung’s disease. Depending on the age of the patient, if there is a normal RAIR, HD is excluded; on the other hand if there is no RAIR, rectal biopsies are required to confirm the diagnosis of HD. For these reasons, it is crucial to be able to perform a reliable test.

In children who are young, uncooperative, anxious, or cognitively and developmentally challenged, it is often impossible to perform this study while awake, and false results may be obtained. In those situations, sedation may be required. Midazolam is frequently the drug of choice, given that it does not influence the presence of RAIR. Often, however, the anxiolytic effect of midazolam is marred by paradoxical agitation or inadequate sedation [8], making the execution of the ARM impossible.

There are no other simple options for sedatives to replace midazolam, and frequently deeper anesthesia is needed. Agents used for deep anesthesia, such as propofol or inhaled agents, can affect gastrointestinal motility and smooth muscle activity, causing a decrease in the anal canal resting pressure [9,10]. Although a recent study demonstrates that in some patients RAIR was elicited only under anesthesia, the preference is for the patient to be responsive and able to follow commands during the study [9,11]. To date, the use of Dexmedetomidine for these procedures has not been adopted because its influence on lower gastrointestinal motility has not been evaluated. Dexmedetomidine is a sedative which was approved by the FDA for adult sedation in 1999. It has not been extensively studied in the pediatric population because it still lacks the FDA approval for children.

Dexmedetomidine (DEX) is a highly selective α2-adrenoceptor agonist that is typically used for sedation. It targets the locus coeruleus, with respiratory-sparing effects. In children, it has been prescribed for procedural sedation, as an adjunct to general and regional anesthesia, as a synergist (with narcotics and anesthetic agents) and as a sedative in the intensive care unit [12,13]. DEX could therefore be utilized as an alternative to midazolam for sedating children for an anorectal manometry study. To our knowledge, the effect of DEX on the IASP and RAIR has never been studied in the pediatric population. The primary objective of this study is to determine the impact of DEX on the IASP and RAIR in order to evaluate if it is a sedative that preserves physiologic anorectal responses and can be offered to children undergoing anorectal manometry.

## 2. Materials and Methods

### 2.1. Study Design and Patient Populations

This was a prospective, open-label study approved by the Boston Children’s Hospital Institutional Review Board and the US Food and Drug Administration (IRB-P00035865; ClinicalTrials.gov: NCT04798482, full protocol accessible; IND130421). This study was conducted under Investigational New Drug (IND) approval #130421, in which the FDA approved the off-label administration of Dexmedetomidine for this specific pediatric population. All data collection occurred in the Gastroenterology Procedure Unit at the Boston Children’s Hospital. The primary objective of this study was to examine the effects of DEX on intra-anal pressure and on the dose–response curve to balloon distention by comparing the measurements taken prior to DEX administration (Baseline) with those recorded at 1 and 5 min after DEX administration.

Patients were eligible to participate if they were 3–18 years old, experiencing chronic constipation, and were scheduled to have an unsedated (while awake) anorectal manometry followed directly by an additional gastrointestinal procedure, which was scheduled to receive deep sedation. The study’s exclusion criteria were as follows:

- Established sedation criteria are not met;
- Patients who require sedation prior to their anal manometry evaluation (with the exception of pre-med midazolam);
- History of allergy, intolerance, or reaction to dexmedetomidine;
- Current, repaired or risk of Moya-Moya disease;
- Recent stroke (cerebrovascular accident) within the past 6 months;
- Uncontrolled hypertension;
- Concomitant use of opioids, beta antagonist, alpha 2 agonist or calcium channel blocker;
- BMI above 30 or weight above the 110th percentile;
- Refusal of IV insertion while awake;
- Currently taking pharmacologic agents for hypertension or cardiac disease;
- Currently taking or has taken digoxin within the past 3 months;
- Active, uncontrolled gastroesophageal reflux (concern for aspiration), requiring endotracheal intubation;
- Current or recent (within the past 3 months) history of apnea requiring an apnea monitor;
- Unstable cardiac status (life threatening arrhythmias, abnormal cardiac anatomy, significant cardiac dysfunction);
- Craniofacial anomaly, which could make it difficult to effectively establish a mask airway for positive pressure ventilation, if required;
- Active, current respiratory concerns that are different from the patient’s baseline status (including pneumonia, exacerbation of asthma, bronchiolitis, respiratory syncytial virus).

Written informed consent to participate in the research study was obtained from a parent/guardian and/or the patient when age-appropriate, prior to the start of the procedure. Once informed consent was obtained, intravenous catheters were initiated as per standard clinical procedure in the GI endoscopy unit. When necessary, children received oral or parenteral midazolam, an anxiolytic established not to affect ARM.

### 2.2. Anorectal Manometry Study Protocol

The anorectal manometry testing was executed using a high resolution catheter and a specialized software (Medtronic, Duluth, GA, USA). Once the patient was in the left lateral decubitus position, a lubricated, standard high resolution manometry catheter was gently inserted into the patient’s anal canal. The catheter was then slowly advanced in the anal canal until the borders of the high-pressure zone (which present the anal sphincter) were identified on the anorectal manometry software screen. The catheter was manually held in place throughout the study to avoid displacement. Once the patient felt comfortable with the presence of the catheter, the intra-anal sphincter pressure (IASP) was identified using a station pull-through, and once the catheter was in the appropriate position, a baseline IASP was obtained over the course of 30 s. To assess the presence of the RAIR, a balloon situated at the proximal end of the catheter was rapidly inflated and deflated with incremental volumes of 10, 20, 30, 40, 50 and 60 mL. Balloon inflations were achieved with a 60 mL syringe that was attached to the catheter probe. After completing the baseline diagnostic ARM evaluation, the catheter remained in the anal canal and 0.5 mcg/kg IV DEX was administered over 1 min followed by an IV infusion of 0.15 mcg/kg/h. The ARM was repeated 1 min after the DEX was administered, and before any other anesthetics were given. During that time, a 30 s baseline was obtained, and progressive balloon inflations of 10, 20, 30, 40, 50 and 60 mL were performed to evaluate for the presence of the RAIR. After 5 min of the infusion, another 30 s baseline was obtained, and progressive balloon inflations of 10, 20, 30, 40, 50 and 60 mL were again performed to evaluate for the presence of the RAIR. The patient was observed for up to 15 min after the start of DEX administration, before the care team continued the patient’s scheduled procedures.

### 2.3. Anorectal Manometry Parameters

The main ARM measurements included resting IASP and the presence and characteristics of the recto-anal inhibitory reflux (RAIR) after the different balloon volumes inflations. Baseline resting IASP was expressed in mmHg and was obtained during a 30 s interval. The presence of RAIR after balloon distension was defined as a decrease of 10% or more between baseline and minimum IASP measurements.

Resting IASP, the presence of RAIR and its characteristics [including the minimum IASP measured during the RAIR, the percent relaxation, the mean latency time (the time between balloon distention and beginning of relaxation), and the duration of relaxation] for each balloon volume inflation were measured pre- and post- DEX infusion (1 and 5 min). The anorectal manometry analysis was performed by a single reviewer who is a pediatric gastroenterology specialist who has extensive experience with motility studies. While performing the analyses, the reviewer was unaware of the patient characteristics, including clinical history and diagnosis.

### 2.4. Dexmedetomidine Protocol

The DEX was administered at 0.5 mcg/kg intravenous bolus followed by a continuous infusion of 0.15 mcg/kg/h until the manometry measurements were completed.

### 2.5. Statistical Analysis

The study was powered and a sample size was calculated assuming a mean intra-anal pressure of 90 mmHg, a mean reduction of 65 mmHg, and a standard deviation of 32. In order to achieve a power of 80.3% at a significance level of 0.05, it was predicted that enrolling approximately 15 subjects would be necessary. Statistical analysis was executed using SPSS Software, version 23.0 (IBM Inc., Chicago, IL, USA). Patient data and procedure-related data are expressed as mean ± standard deviation. A student *t*-test was utilized to evaluate statistical significance. A *p* value of <0.05 was considered to be statistically significant.

## 3. Results

### 3.1. Patient Population

A total of 20 children between 3 and 18 years old (mean age 10.8 ± 4.6 years, 60% female, ASA score < 3) were included in the study. Two children presented with already-diagnosed Hirschsprung disease (HD). One child with HD and another with anal achalasia had received chemical denervation to the IAS with botulinum toxin prior to ARM (16 months and 38 months prior to ARM, respectively).

### 3.2. Intra-Anal Resting Pressure

**Figure 1:**
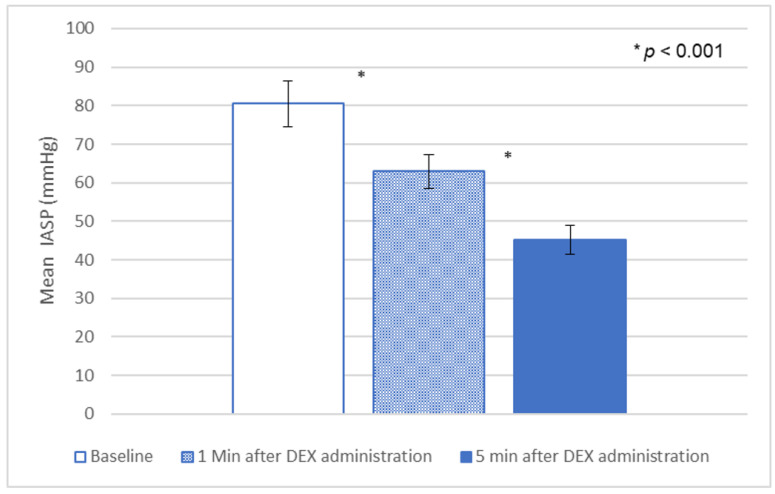
Dexmedetomidine effect on resting IASP.

There was a statistically significant decrease (*p* < 0.001) in IASP when comparing the values before and after the administration of DEX (Figure 1). The mean resting intra-anal sphincter pressure (IASP) had decreased from the baseline of 80.5 ± 5.9 mmHg to 62.9 ± 4.5 mmHg and 45.1 ± 3.7 mmHg (at 1 min and 5 min after DEX administration, respectively).

### 3.3. Recto-Anal Inhibitory Reflex Presence

At baseline before DEX administration, the RAIR was present in 16 patients, and absent in four patients. Of the four patients with an absent RAIR at baseline, two had known Hirschsprung disease. The other two patients had normal rectal biopsies that showed presence of ganglion cells, providing the diagnosis of IAS achalasia. In all four patients, RAIR was absent both before and after the administration of Dexmedetomidine. In 2/16 (12.5%) of the patients for whom RAIR was present at baseline, the RAIR became absent after DEX administration, one after 1 min, and the second one after 5 min.

### 3.4. Recto-Anal Inhibitory Reflex Characteristics

**Figure 2:**
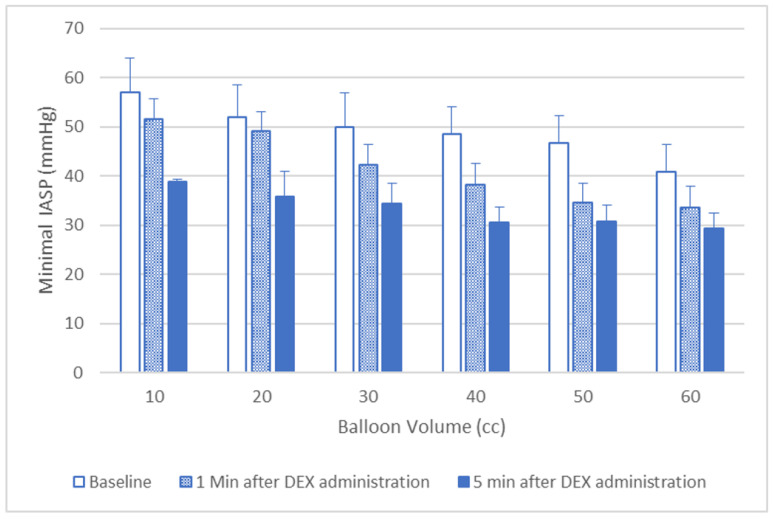
Dexmedetomidine effect on minimal IASP after balloon inflation. In all cases there was a statistically significant difference (*p* < 0.05) between baseline and DEX administration.

**Figure 3:**
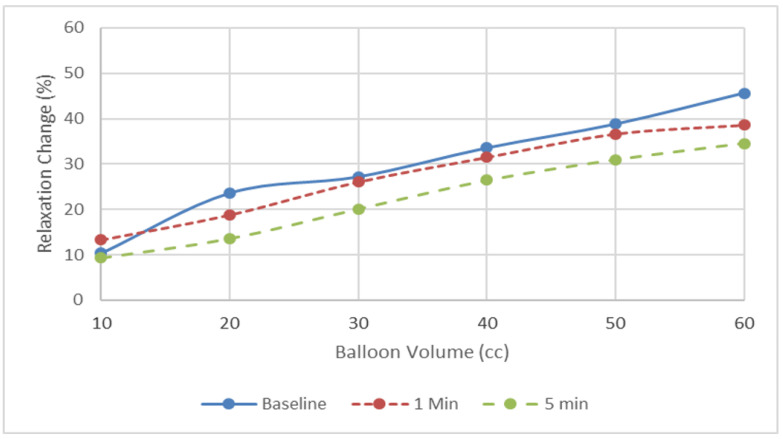
Dexmedetomidine effect on relaxation change after balloon inflation. There was a tendency for the dose–response curve to shift to the right, but there were no significant differences.

The minimum IASP measured throughout the RAIR for all balloon volumes were lower during DEX administration, compared to baseline (Figure 2). The percentage of relaxation change after balloon distension was lower during DEX administration, compared to baseline, with balloon volume of 20 mL and above (Figure 3). This decrease in relaxation change was more profound after 5 min compared to 1 min. No difference was observed over the latency time or the total relaxation time for any of the balloon volumes before and after DEX administration (Table 1).

## 4. Discussion

Constipation is a frequently reported problem in the pediatric population and is a common reason for a pediatrician visit. Oftentimes, the etiology for the constipation is functional and is diagnosed based on a patient’s presenting symptoms. However, in some cases, a diagnostic work-up is required, and in severe, intractable cases, motility studies are necessary. The most frequently used motility study in children is the anorectal manometry study, which is typically performed while the child is awake. However, in children who are quite young, uncooperative, or anxious, the efficient and accurate execution of anorectal manometry testing may be challenging and receiving an adequate measurement may be difficult. In these circumstances, providers will often elect to use anesthetics, and thus, it is important to know the effect they might have on the study results.

In the present study, we show that administering DEX as a sedative in patients undergoing anorectal manometry has significant effects on anorectal function, and thus may also affect an ARM study’s results and conclusions. We show that after DEX administration, the intra-anal pressure significantly decreased. Additionally, we observed significant changes in the RAIR, with a decrease in the amount of relaxation after balloon distention. In two patients, the RAIR was considered lost. Given that anorectal manometry is considered to be one of the most frequently employed motility studies performed in pediatrics, and that at times it is necessary to use sedation, it appears that DEX has some limitations that need to be considered prior to its use [8].

The anal sphincter consists of two muscular components, an external and an internal anal sphincter. The internal anal sphincter is derived from the circular muscle layer of the rectum, and is composed primarily of smooth muscle. Previous studies have shown that the internal anal sphincter contributes to the majority of the resting IASP (75–85%) [14], and, therefore, it is expected that general anesthesia will influence the anal sphincter function and its relaxation [15].

Midazolam is generally the preferred medication when sedation for ARM is required [4,8]. This is because it produces a relaxing effect by acting on glycine receptors, yet it does not change the resting IASP or the presence of RAIR [8]. However, in some cases, the sedative effects of midazolam are inadequate for adequately performing the ARM procedure [9,11]. Therefore, other alternatives are needed. A study that evaluated the impact of ketamine during ARM concluded that it did not affect the IASP or the presence of the RAIR [16,17]; however, ketamine has been associated with behavioral reactions, agitation, hallucinations, vomiting and longer recovery times in children, and thus, is rarely utilized.

Propofol is a short-acting lipophilic intravenous agent that is used extensively for anesthesia induction and maintenance. Propofol is known to have a relaxing effect on smooth muscles throughout the body, and we have previously shown that propofol significantly decreases IASP pressure, but still allows the detection of the RAIR [9]. It has also been shown that in some instances when the children are very uncooperative and it is not possible to elicit the RAIR while awake, a normal RAIR can be demonstrated once the patient receives propofol. A recent study retrospectively evaluated the impact of various general anesthetic agents on the detection of RAIR and on the resting IASP during an anorectal manometry study [11]. In this study, it was found that sevoflurane, propofol, nitrous oxide and fentanyl, alone or in combination, all affected resting IASP [8,9,11] by decreasing it. It was shown that in all cases, it was still possible to detect the RAIR. From these studies, it is clear that the use of inhalational agents or propofol can be used to detect the presence of the RAIR, but cannot be used to obtain information on the basal IASP. This parameter is important when evaluating children with incontinence, or in those with HD in which the application of Botox is being considered.

Thus, alternative sedation options are needed. DEX is a highly selective a2-adrenoceptor agonist, and therefore has a different mechanism of action unlike anesthetics, narcotics and volatile anesthetics. It has sedative, analgesic and opioid-sparing effects, which deems it a suitable sedation option for some procedures, particularly those which require minimal analgesia. DEX has good bioavailability by the intramuscular (104%), intravenous, intranasal (65%) and buccal (82%) route. It has poor bioavailability by the oral route (16%) and would not be a recommended route of administration [18,19]. As an alpha 2 agonist with sedative properties, DEX has been shown to produce EEG activity which mirrors natural stage 2 or 3 of non-REM sleep [20]. DEX does not have a direct effect on the medulla, eliciting sedation via pathways between the locus coeruleus to posterior cingulate cortex, thalamus and basal ganglia [21,22]. DEX does not have a direct effect on respiratory drive and preserves airway reflexes [23]. The sedative effect of DEX is dose-dependent at lower plasma serum levels. Studies have shown that the bispectral index and hemodynamic responses (blood pressure and heart rate), are correlated with the plasma serum concentration of DEX [24]. The potential for using DEX to perform ARM studies has often been encouraged as a subject for clinical investigation, but to date had never been evaluated.

In our study we demonstrated that DEX was effective and safe in allowing the performance of the ARM. The administration of DEX was associated with significantly decreases the IASP with lower values observed as the length of administration of the drug increased (Figure 1 and Figure 2). We also show it might have an effect on the results of the anorectal manometry, causing the loss of RAIR in 12.5% of our patients who had normal RAIR present when they were awake. This usually occurred in the latter periods of the administration of the drug. Additionally, we showed that DEX had a tendency to push the dose–response curve to the right, indicating that the RAIR evaluation during DEX administration cannot be used to exclude spinal cord abnormalities, in which there is usually a leftward push of the dose–response curve of IAS relaxation after various balloon volumes.

There have been some studies examining DEX on upper gastrointestinal tract function. Additionally, there been studies in adult patients that have shown that DEX does impact upper esophageal sphincter function with pharyngeal swallowing and depressed the contractile function of the proximal esphagus with esophageal swallowing [25]. In animal studies, DEX has shown to have a dose-dependent effect on the inhibition of gastrointestinal motility (duodenum, jejunum, colon and cecum) in donkeys [26]. However, the effects on the lower gastrointestinal tract in humans suggest that DEX may have a positive effect on improving return of motility following abdominal surgery in adult patients [27]. Results of animal experiments have suggested that α-adrenoceptor mediated pathways may play a role in modulating smooth muscle motility, but mixed effects have been reported [15,17,25,28,29].

There are no published studies examining the effect of DEX on the human IAS. A recent study on healthy adult human volunteers demonstrated a significant decrease in both resting and relaxation pressures recorded at the gastroesophageal junction [25]. These findings are similar to what we observed here on the effects of Dexmedetomidine on IAS, suggesting that α2-activation is associated with decreased sphincter pressure. Given the different sub-variants of α2-adrenoceptor receptors [25] it is not surprising that some animal studies have also previously described that α2-adrenoceptors can at times mediate contraction of the IAS [15,17]. These occasional contractions can explain our observation that in two patients the RAIR disappeared while on DEX. This might also explain our finding that the dose–response curve to the rectal balloon distention was shifted to the right, implicating that the decrease in IASP was lower under DEX effect compared to the change in pressure while awake.

As mentioned, this study also exhibited a significant decrease in IAP after DEX administration in all subjects, including in those who were known to have HD. This is important when the baseline IAS pressure is being used to understand the pathophysiology of symptoms, such as in HD patients with obstructive symptoms, or to make a decision regarding therapeutic interventions (for example, Botox administration) [29,30,31].

There are some limitations to our study. First, our patient population consisted of pediatric patients with severe defecation symptoms, so a comparison with healthy controls was not possible. Additionally, the present study was at a single center and was limited by its small sample size. In the future, additional studies should be performed with a larger and more diverse patient population.

## 5. Conclusions

In conclusion, we have shown for the first time that DEX significantly decreases IASP, and seems to shift the dose–response curve of the RAIR to the right, producing a loss of RAIR. Therefore, DEX should be used cautiously during ARM to exclude a non-relaxing IAS. DEX should also not be used to when evaluating a baseline IAP if a therapeutic intervention is being considered, as the pressure is significantly reduced during its administration.

## Figures and Tables

**Table 1:** Mean latency time and total relaxation times (s).

	Latency Time				Relaxation Time			
Balloon Volume (mL)	Baseline	1 min after DEX Administration	5 min after DEX Administration	*p* Value	Baseline	1 min after DEX Administration	5 min after DEX Administration	*p* Value
10	2.72 ± 0.78	2.54 ± 1.73	2.13 ± 1.39	0.4	6.98 ± 5.07	8.61 ± 2.30	6.07 ± 0.9	0.3
20	2.4 ± 1.14	3.75 ± 3.01	2.6 ± 3.11	0.3	9.4 ± 5.66	8.18 ± 3.82	6.47 ± 2.28	0.2
30	3.36 ± 0.7	3.43 ± 1.20	2.24 ± 1.7	0.09	11.51 ± 6.17	9.38 ± 4.87	9.74 ± 7.15	0.6
40	3.55 ± 0.8	3.08 ± 1.34	3.61 ± 1.68	0.1	13.73 ± 7.77	10.79 ± 7.29	10.47 ± 6.52	0.3
50	3.67 ± 1.5	3.44 ± 1.62	2.66 ± 1.51	0.08	14.61 ± 7.93	16.04 ± 10.26	17.75 ± 14.9	0.5
60	3.45 ± 1.6	3.25 ± 1.12	3.02 ± 1.28	0.2	16.62 ± 14.5	14.37 ± 6.75	12.19 ± 5.25	0.4

## Data Availability

The data presented in this study are available on request from the corresponding author. The data are not publicly available due to confidentiality.
